# Two Expansin Genes, *AtEXPA4* and *AtEXPB5*, Are Redundantly Required for Pollen Tube Growth and *AtEXPA4* Is Involved in Primary Root Elongation in *Arabidopsis thaliana*

**DOI:** 10.3390/genes12020249

**Published:** 2021-02-10

**Authors:** Weimiao Liu, Liai Xu, Hui Lin, Jiashu Cao

**Affiliations:** 1 Laboratory of Cell and Molecular Biology, Institute of Vegetable Science, Zhejiang University, Hangzhou 310058, China; 11616044@zju.edu.cn (W.L.); 11416052@zju.edu.cn (L.X.); 2 Key Laboratory of Horticultural Plant Growth, Development and Quality Improvement, Ministry of Agriculture, Hangzhou 310058, China; 3 Crop Research Institute, Fujian Academy of Agricultural Sciences, Fuzhou 350013, China; lhlzl540@163.com; 4 Zhejiang Provincial Key Laboratory of Horticultural Plant Integrative Biology, Hangzhou 310058, China

**Keywords:** *Arabidopsis thaliana*, expansin genes, *AtEXPA4*, *AtEXPB5*, pollen tube growth, root elongation

## Abstract

The growth of plant cells is inseparable from relaxation and expansion of cell walls. Expansins are a class of cell wall binding proteins, which play important roles in the relaxation of cell walls. Although there are many members in expansin gene family, the functions of most expansin genes in plant growth and development are still poorly understood. In this study, the functions of two expansin genes, *AtEXPA4* and *AtEXPB5* were characterized in *Arabidopsis thaliana*. *AtEXPA4* and *AtEXPB5* displayed consistent expression patterns in mature pollen grains and pollen tubes, but *AtEXPA4* also showed a high expression level in primary roots. Two single mutants, *atexpa4* and *atexpb5*, showed normal reproductive development, whereas *atexpa4* *atexpb5* double mutant was defective in pollen tube growth. Moreover, *AtEXPA4* overexpression enhanced primary root elongation, on the contrary, knocking out *AtEXPA4* made the growth of primary root slower. Our results indicated that *AtEXPA4* and *AtEXPB5* were redundantly involved in pollen tube growth and *AtEXPA4* was required for primary root elongation.

## 1. Introduction

Plant growth and development are inseparable from cell proliferation and expansion. Different from animal cells, plant cells are surrounded by cell walls, which are highly dynamic and complex networks [1]. Cells are always in a dynamic balance between the expansion of protoplasts and the restraint of cell walls, undergoing irreversible growth [2]. Although cell walls expand slowly while plants grow, some organs and tissues require rapid expansion of cell walls during specific periods, such as fast-growing roots and pollen tubes.

Numerous cell wall synthesis and remodeling genes have been reported to be involved in cell wall expansion [3,4,5,6,7,8,9], including expansin genes. Expansins can weaken non-covalent bonds between cell wall polysaccharides, thereby promoting slippage and relaxation of cellulose microfibers, resulting in cell wall extension and cell expansion [10,11]. Expansins usually have two typical domains, DPBB_1 and Pollen_allerg_1, and most of expansins have signal peptides [12]. DPBB_1 domain is often related to family-45 glycosyl hydrolase (GH45), and contains a conserved region that has the double-psi beta-barrel (DPBB) fold [13,14]. Although DPBB_1 is similar to GH45, expansins lack the β-1,4-glucanase activity of GH45 enzymes, which in turn lacks the wall expanding activity of expansins [15]. Pollen_allerg_1 is often a pollen allergen, which is found at the C-terminus of expansins [16]. The expansin gene family with many members can be divided into two major subfamilies, namely EXPA and EXPB subfamilies [15]. The various temporal and spatial expression patterns of expansin genes imply that they perform corresponding functions in different developmental stages [12,17,18,19]. Many studies have shown that EXPA subfamily plays complex and diverse functions in plant growth and development. For example, *Arabidopsis thaliana EXPA2* (*AtEXPA2*) participates in seed germination under the regulation of upstream transcription factors [20,21,22]. *AtEXPA5* participates in plant growth and development regulated by ethylene and brassinosteroids [23,24]. *AtEXPA5* and *Oryza sativa EXPA8* (*OsEXPA8*) [25,26] are both involved in the development of primary roots. The inhibition of their expressions reduces the length of primary root. Moreover, *AtEXPA14* and *AtEXPA17* participate in the growth of lateral roots and root hairs by the regulation of auxin-related transcription factor LBD18 [27,28].

However, researches on the roles of expansins in plant reproductive development are relatively limited. It was only confirmed that *Zea mays* EXPB1 (ZmEXPB1) showed a great influence on the growth of pollen tubes in vivo and positively affected the entry of pollens into ovules [29,30,31].

A previous study showed that two expansin genes, *AtEXPA4* and *AtEXPB5*, were strongly expressed in dry pollen grains, imbibed pollen grains, and pollen tubes [32,33,34,35,36]. Here, we further confirmed the expression patterns of *AtEXPA4* and *AtEXPB5*. By exploring phenotypes of mutants and overexpression lines, we found that *AtEXPA4* and *AtEXPB5* are redundantly involved in pollen tube elongation, and *AtEXPA4* plays a positive role in primary root growth.

## 2. Materials and Methods

### 2.1. Plant Materials and Growth Conditions

All transgenic plants used in this study were of *Arabidopsis thaliana* (*A. thaliana*) Columbia ecotype (Col-0) background and were obtained by *Agrobacterium tumefaciens*-mediated floral dip method [37]. The homozygous mutants *atexpa4* and *atexpb5* were obtained by CRISPR/Cas9 system [38]. Two off-target sites of *AtEXPA4* and *AtEXPB5* were predicted by the CRISPR-P 2.0 website (http://crispr.hzau.edu.cn/CRISPR2/ (accessed on 1 February 2020)), respectively. To obtain heterozygous double mutants of *AtEXPA4* and *AtEXPB5*, *atexpb5* and *atexpa4* homozygous plants were used as female and male parents in a cross. The homozygous double mutants, *atexpa4expb5*, were generated by self-crossing with heterozygous F_1_ plants. The genotypes of 362 F_2_ plants were confirmed by PCR and sequencing, and genotype statistics and analysis were performed.

A 1522-bp promoter sequence and the coding sequence of *AtEXPA4* (splicing variant: At2g39700.1) were amplified from Col-0 genomic DNA and inflorescence cDNA. A 1571-bp promoter sequence and the coding sequence of *AtEXPB5* (splicing variant: At3g60570.1) were also amplified from Col-0 genomic DNA and inflorescence cDNA. Then they were subcloned into pBI101 vectors to create the fusion overexpression constructs *proAtEXPA4::EXPA4*, *proAtEXPB5::EXPB5* and the promoter analysis construct *proAtEXPA4::GUS*, *proAtEXPB5::GUS*. The complemented lines were obtained by transforming *proAtEXPA4::EXPA4* and *proAtEXPB5::EXPB5* into the double mutant *atexpa4expb5*, and named *atexpa4expb5^A4OE^* and *atexpa4expb5^B5OE^*, respectively. The homozygous overexpression lines *AtEXPA4^OE^, AtEXPB5^OE^* and promoter analysis lines of *AtEXPA4*, *AtEXPB5* were identified from screening T_3_ plants on plates containing kanamycin (50 mg·L^−1^). Two complementary lines were identified from screening T_1_ plants on plates containing kanamycin (50 mg·L^−1^). All seedlings were transplanted into soil, and grown in a 22 °C climate chamber under long-day conditions (16 h light/8 h dark). All the primers were listed in Table S1.

For root phenotype analysis, the seeds of wild-type, *atexpa4*, *atexpb5, AtEXPA4^OE^*, and *AtEXPB5^OE^* were surface-sterilized with 75% ethanol for 5 min and washed 5 times with sterile water. Subsequently, these seeds were geminated on 1/2 Murashige and Skoog solid media and placed horizontally at 4 °C for 3 days. Next, plates were transferred to an artificial climate chamber and placed vertically on the platform, and then germinated at 22 ± 1 °C under 12 h light/12 h dark cycle.

### 2.2. mRNA Expression Analysis

Trizol reagent (Invitrogen, Carlsbad, CA, USA) were used to extract total RNA from plant tissues and first-strand cDNA was synthesized by PrimerScript RT reagent Kit (TaKaRa, Kyoto, Japan) from 1 μg total RNA. The relative expression levels of corresponding genes in different tissues and organs were detected by quantitative real-time PCR (qRT-PCR), which were performed by using TaKaRa TB Green™ Premix Ex Taq™ II (Tli RNaseH Plus) on a Real-Time PCR machine (CFX96 Real-Time System, Bio-Rad, Carlsbad, CA, USA). Primers for *AtEXPA4* and *AtEXPB5* were listed in Supplemental Table S1. *BETA-TUBULIN4* (At5g44340) was used as the reference gene for *AtEXPA4* and *AtEXPB5* expression in different tissues and different transgenic lines. All experiments were performed three biological replicates, and each biological replicate was performed three technical replicates. Moreover, transcript levels of target genes were calculated relative to *BETA-TUBULIN4* using the 2^−ΔΔCt^ method.

### 2.3. Histochemical GUS Staining Assay to Analyze Promoter Activity

The promoter analysis constructs, *proAtEXPA4::GUS* and *proAtEXPB5::GUS*, were transferred into wild-type plants by *Agrobacterium tumefaciens*-induced floral dip method. More than six independent T_1_ lines were screened continuously until T_3_ homozygous plants were obtained. Seedlings and inflorescences from homozygous plants were used to detect GUS activity. The inflorescences from 35-day-old plants and seedlings at different stages were stained with GUS working solution (GUS working solution: X-Gluc solution and Basic solution (1:9, *v*/*v*). X-Gluc solution: dissolving X-Gluc with N-N-dimethylamide (DMF) to make a 20 mM solution. Basic solution: 50 mM NaH_2_PO_4_, 50 mM Na_2_HPO_4_, 10 mM Na_2_EDTA, 0.1% (*v*/*v*) Triton X-100, 0.5 mM K_3_ [Fe(CN)_6_]), and 0.5 mM K_4_ [Fe(CN)_6_].) and then incubating in dark at 37 °C overnight. Tissues were decolorized in 75% and 90% ethanol in order, and images were taken by a differential interference microscopy (Nikon, Tokyo, Japan). Each tissue staining contained at least three biological replicates. Moreover, the stage of flower development was confirmed by previous studies [39,40].

### 2.4. Subcellular Localizations of AtEXPA4 and AtEXPB5

To observe subcellular distributions of AtEXPA4 and AtEXPB5, we amplified the *AtEXPA4* and *AtEXPB5* coding sequences with gene-specific primers (Table S1), and then subcloned these into a vector containing the enhanced green fluorescent genes (*eGFP*), to form *pro35S*::*AtEXPA4*::*eGFP* and *pro35S*::*AtEXPB5*::*eGFP* constructs. The fusion vectors were transiently transformed into onion epidermal cells using a helium-driven accelerator (PDS/1000, Bio-Rad). The parameters were as follows: 1 μm gold particles, 1100 psi bombardment pressure, and a distance of 9 cm from microcarrier to samples. After 24 h of cultivation, the onion epidermal cells with *eGFP* expression were observed and pictures were taken by a fluorescence microscope (ECLIPSE 90i, Nikon). To visualize eGFP distribution, onion epidermal cells were plasmolyzed in 0.1 g·mL^−1^ sucrose solution for 5 min. For subcellular localization experiments using tobacco (*Nicotiana benthamiana*), the fusion vectors were transiently transformed into leaf epidermal cells by the infiltrated method. After 48 h of introduction, the subcellular localization of eGFP-fusion protein was analyzed by a confocal laser scanning microscope (A1, Nikon) with the NIS-elements AR software version 4.60(Nikon).

The subcellular localization experiments of AtEXPA4 and AtEXPB5 have been carried out using at least three biological replicates, and no less than 5 cells with eGFP signal were observed in each replicate.

### 2.5. Analysis of Primary Root and Meristem Length

To observe primary root growth, the 3, 5, 7-day-old seedlings were taken photos by a stereo microscope (Leica, Germany). Subsequently, the primary root length was measured ranging from the base of hypocotyl to the root tip by ImageJ 1.52a software (https://imagej.nih.gov/ij/ (accessed on 29 March 2020)).

The length of root meristem was defined as the distance from quiescent center (QC) to transition zone (TZ, that is the position of the first elongating cortical cell) [4]. To measure the length of root meristems, roots were fixed in saturated chloral hydrate and images were taken by a differential interference microscopy (Nikon, Japan). All experiments were performed in three biological replicates with at least 12 seedlings measured in each replicate.

### 2.6. Phenotype Analysis of Pollen and Pollen Tube

Alexander staining was used to detect pollen vitality [41]. Mature pollen grains that develop normally will be dyed red or purple, and abnormal or immature pollen grains will be dyed blue or green. 4′,6-Diamidino-2-phenylindole (DAPI) staining was used to detect whether pollen nuclei were normal [42]. Under ultraviolet light, normal pollen nuclei (two sperm nuclei and one nutrient nucleus) will produce intense fluorescence. Aniline blue staining could detect callose development and degradation [43]. In tetrad stage, microspores are wrapped by callose wall, which can combine with the aniline blue dye to produce strong fluorescence under ultraviolet illumination. Under normal circumstances, the callose wall gradually degrades with the development of pollen. In mature pollen stage, the callose wall has been completely degraded and only the weak fluorescence of pollen grains can be observed but no strong fluorescence will appear under ultraviolet light. All micrographs were taken by an inverted fluorescence microscope (Nikon, Japan). Scanning electron microscope (SEM) observation and pollen germination in vivo were conducted as described in Lin et al. [44]. The mature pollens and tetrads were peeled off from flower buds in stage 13 and stage 6–8 of 35-d-old inflorescences, respectively. Each kind of staining was repeated three times, using at least 5 plants each time, and observing no less than 30 fields of view. The length of pistil and pollen tube was measured by ImageJ, and the ratio of pollen tube to pistil length was calculated as an indicator of the germination degree of pollen tube. The experiments were carried out using three biological replications with at least 7 pistils measured in each replicate.

## 3. Results

### 3.1. Analysis of AtEXPA4 and AtEXPB5 Expression Patterns

**Figure 1:**
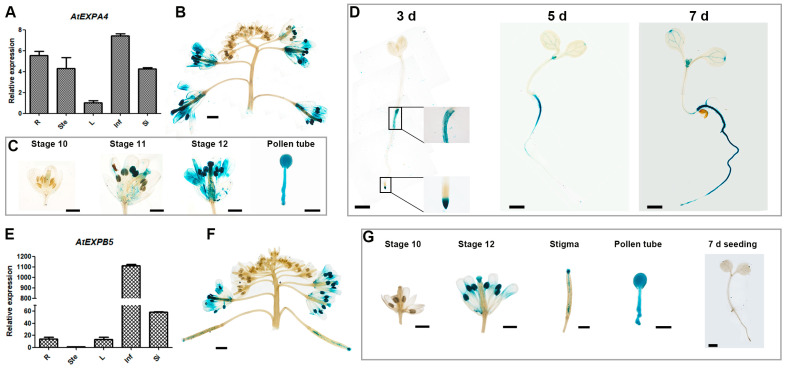
Expression pattern analysis of *AtEXPA4* and *AtEXPB5*. (**A**,**E**) qRT-PCR analysis of *AtEXPA4* (**A**) and *AtEXPB5* (**E**) transcripts in different tissues of *Arabidopsis thaliana*: 35-d-old roots (R), 35-d-old stems (Ste), young rosette leaves (L), 35-d-old inflorescences (Inf) and siliques (Si). *BETA-TUBULIN4* was used as the reference gene. *AtEXPA4* expression in leaf and *AtEXPB5* expression in stem were normalized to 1. The values are the mean ± SD (standard deviation), three biological replicates with three technical replicates in each biological replicate. (**B**–**D**) Analysis of *proAtEXPA4*::*GUS* activity. (**B**) GUS activity in inflorescences. (**C**) GUS activity in different developmental stages of buds and pollen tubes. (**D**) GUS activity in seedlings at different time points after germination, days (d). (**F**,**G**) Analysis of *proAtEXPB5*::*GUS* activity. (**F**) GUS activity in inflorescences. (**G**) GUS activity in different developmental stages of buds, stigmas, pollen tubes, and 7-d-old seedlings. Scale bars of pollen tubes, 25 μm. Scale bars of others, 1 mm.

Through the analysis of domains on the Pfam website (http://www.sanger.ac.uk/Software/Pfam/ (accessed on 21 March 2018)), we confirmed that both AtEXPA4 and AtEXPB5 contain DPBB_1 and Pollen_allerg_1, which have typical domains of the expansin gene family (Figure S1) [12].qRT-PCR and GUS reporting system were used to explore spatial and temporal expression of them. The results showed that *AtEXPA4* seemed to expressed ubiquitously (Figure 1A), but its expression level was higher in inflorescences and roots (Figure 1A,B). In inflorescences, *AtEXPA4* was expressed from the stage 11 of floral buds to the mature pollen stage. Subsequently, strong GUS signal appeared in pollen tubes, which indicated that *AtEXPA4* was also highly expressed during pollen tube growth (Figure 1C). Further research found that *AtEXPA4* was expressed in the root tips, especially in root meristems of 3-d-old seedlings. As time passed, *AtEXPA4* was expressed in the entire root by the 7th day after seed germination (Figure 1D). As for *AtEXPB5*, the qRT-PCR result showed that *AtEXPB5* was predominantly expressed in inflorescences and siliques, but weakly expressed in other tissues (Figure 1E,F). GUS staining also confirmed that *AtEXPB5* was highly expressed in inflorescences. Moreover, GUS signal in pollen tube was very significant, suggesting that *AtEXPB5* was strongly expressed during pollen tube growth (Figure 1G).

### 3.2. AtEXPA4 and AtEXPB5 Exhibit Cell Wall Localizations

**Figure 2:**
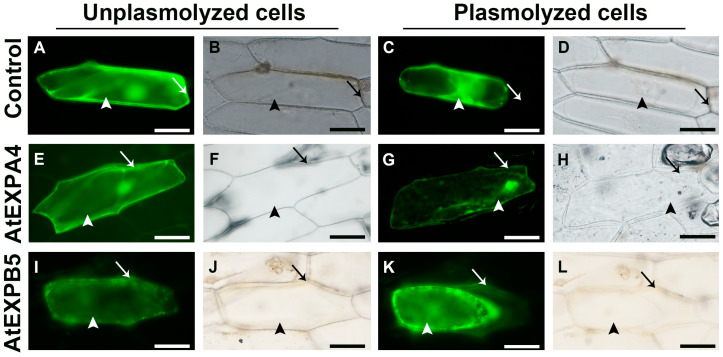
Subcellular localizations of AtEXPA4::eGFP and AtEXPB5::eGFP fusion proteins in onion epidermal cells. (**A**–**D**), Control cells with eGFP signals. (**E**–**H**), Onion epidermal cells with AtEXPA4::eGFP fusion signals. (**I**–**L**), Onion epidermal cells with AtEXPB5::eGFP fusion signals. (**A**,**C**,**E**,**G**,**I**,**K**), Fluorescence images. (**B**,**D**,**F**,**H**,**J**,**L**), Brightfield images. (**A**,**B**,**E**,**F**,**I**,**J**), Unplasmolysed cells. (**C**,**D**,**G**,**H**,**K**,**L**), Plasmolysed cells. Arrows and arrow heads indicate cell walls and cytoplasm, respectively. Scale bars, 100 μm.

To test whether AtEXPA4 and AtEXPB5 were cell wall binding proteins, *eGFP* genes were fused to *AtEXPA4* and *AtEXPB5* coding sequences to form *AtEXPA4*::*eGFP* and *AtEXPB5*::*eGFP* constructs under the control of constitutive *CaMV 35S* promoter. Subsequently, these constructs were transiently transformed into onion and tobacco epidermal cells. The eGFP fluorescence in tobacco leaf epidermal cells appeared in nuclei and plasma membrane, which indicated that AtEXPA4 and AtEXPB5 localized in nuclei and plasma membrane (Figure S2). Furthermore, the eGFP fluorescent signal associated with AtEXPA4 and AtEXPB5 localized homogeneously in unplasmolyed transformed onion cells, including cytoplasm and nuclei (Figure 2E,F,I,J). In plasmolyed cells, the eGFP fluorescence not only appeared in cytoplasm, nuclei, and cell membrane, but also distributed on the cell wall (Figure 2G,H,K,L). On the contrary, there was no eGFP signal on the cell wall in the control cells after plasmolysis (Figure 2C,D). These results confirmed that AtEXPA4 and AtEXPB5 exhibited cell wall localizations.

### 3.3. Identification of atexpa4, atexpb5, atexpa4expb5 Mutants, and AtEXPA4^OE^, AtEXPB5^OE^ Lines

**Figure 3:**
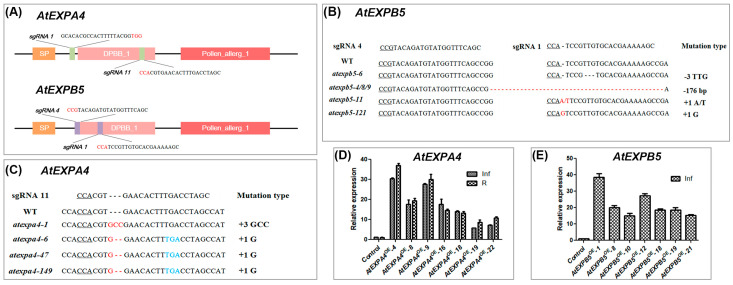
Confirmation of *AtEXPA4* and *AtEXPB5* transgenic plants. (**A**), Genomic location of sgRNAs targeting to *AtEXPA4* and *AtEXPB5*. PAM sites are highlighted in red. (**B**,**C**), the gene editing situation of *AtEXPA4* and *AtEXPB5* by CRISPR/Cas9 system in T_3_ plants. Underlined letters, red letters, and blue letters represent PAM sites, gene editing sites, and stop codons, respectively. (**D**), qRT-PCR analysis of *AtEXPA4* expression in *AtEXPA4^OE^* lines. 35-d-old roots (R) and inflorescences (Inf). *BETA-TUBULIN4* was used as the reference gene. *AtEXPA4* expression in control lines were normalized to 1. (**E**), qRT-PCR analysis of *AtEXPB5* expression in *AtEXPB5^OE^* lines. 35-d-old inflorescences (Inf). *BETA-TUBULIN4* was used as the reference gene. *AtEXPB5* expression in control lines were normalized to 1. The values are the mean ± SD, three biological replicates with three technical replicates in each biological replicate.

In order to characterize the functions of *AtEXPA4* and *AtEXPB5* in plant growth and development, gene knockout and overexpression methods were used. The promoter of *Cas9* was replaced with *YAOZHE* promoter (*proAtYAO*) for improving the editing efficiency of CRISPR/Cas9 system (Figure S3A) [45]. Two sgRNAs targeting *AtEXPA4* or *AtEXPB5* were designed respectively to obtain knockout lines. For *AtEXPB5*, both sgRNAs worked, while only sgRNA11 of *AtEXPA4* worked (Figure 3A and Figure S4). Subsequently, homozygous lines *atexpa4*-*6*/*47*/*149* and *atexpb5*-*4*/*8*/*9* were used for phenotypic observation (Figure 3B,C). We also checked whether these lines were off-target by PCR and sequencing methods. The results showed that none of the 33 *atexpa4* and 47 *atexpb5* plants at off-target sites were edited (Tables S2 and S3). Moreover, homozygous double mutant *atexpa4expb5* was screened by PCR and sequencing (Figure S5). In addition, *AtEXPA4* and *AtEXPB5* were overexpressed under the control of their own promoters (Figure S3B,C). For *AtEXPA4^OE^*, seven overexpression lines were generated and they all showed 5.8–37.1-fold increase in roots and inflorescences compared with wild-type.

As for *AtEXPB5^OE^*, a total of 7 overexpression lines were obtained, which showed an increase of 15.0–38.5-fold compared with the wild type. The growth of homozygous lines with the highest overexpression, *AtEXPA4^OE^*-4 and *AtEXPB5^OE^*-1 were subsequently monitored (Figure 3D,E).

### 3.4. Varied Expressions of AtEXPA4 and AtEXPB5 Show No Effect on Plant Morphology and Pollen Vitality

The results of morphological observation showed that whether *AtEXPA4* and *AtEXPB5 were* overexpressed or knocked out, plant overall morphology was not affected. Moreover, sepals, petals, stamens and pistils were not significantly abnormal compared with wild-type. Also, *double mutant, atexpa4expb5*, was not different from control lines (Figure S6).

Furthermore, pollen development of transgenic plants was observed by cytological staining. Alexander staining revealed that pollen grains produced by these transgenic plants all showed high vitality. By aniline blue staining, they all showed normal thickening of callose during the tetrad stage, and no abnormality in callose degradation during the subsequent pollen maturity stage. DAPI staining was used to observe pollen nuclei, and the result displayed that all transgenic pollen nuclei were development normally. SEM observation revealed that pollen morphology and surface decoration of all transgenic lines were no different from those of wild-type. (Figure S7). These results indicated that the single and double mutations and overexpression of *AtEXPA4* and/or *AtEXPB5* had no effect on pollen development.

### 3.5. AtEXPA4 and AtEXPB5 Are Redundantly Required for Pollen Tube Growth

**Figure 4:**
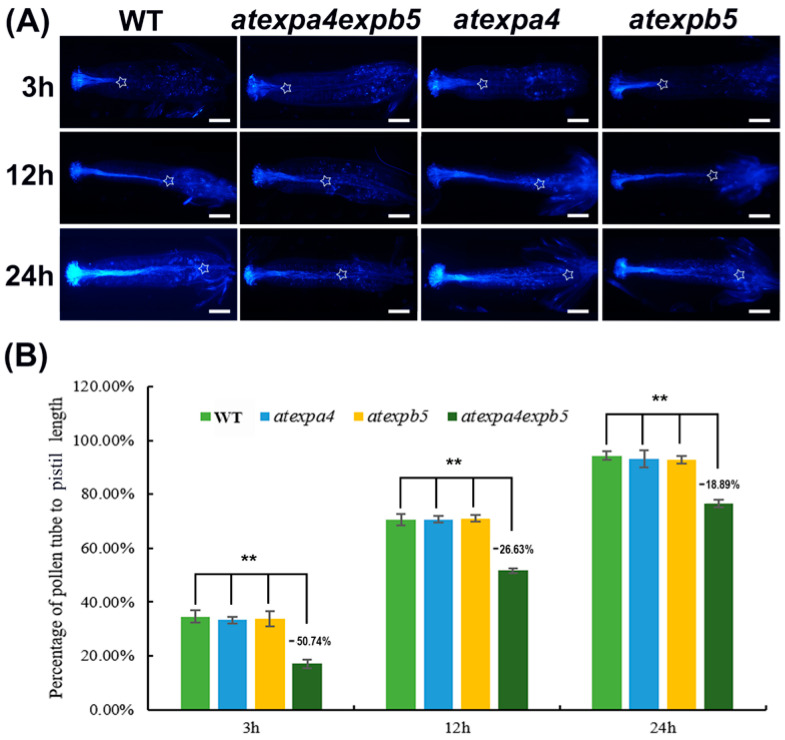
*AtEXPA4* and *AtEXPB5* are involved in pollen tube growth. (**A**), Aniline blue staining of pollen tubes at 3 h, 12 h, and 24 h after pollination in single and double mutants, hours (h). Asterisks show positions where pollen tubes arrive, scale bars, 400 μm. (**B**), the percentage of pollen tube to pistil length in (**A**). Values are means, error bars are SD, *n* = 12 pistils per replicate, three biological replicates, *t*-tests as ** *p* < 0.01.

To investigate the impacts of *AtEXPA4* and *AtEXPB5* on pollen germination, in vivo germination assay was performed. After 3 h of pollen germination, wild-type pollen tube length accounted for 34.59% of total pistil length, while the pollen tube length of *atexpa4expb5* only accounted for 17.04% of pistil length. The pollen tube length of *atexpa4expb5* was 50.74% less than that of wild-type (Figure 4A,B). With pollen tube development, the differences in pollen tube length between double mutants and wild-type became smaller than that in the 3 h after pollen germination, but they were still at significant levels. (Figure 4B). By 24 h after pollination, wild-type pollen tubes have been extended to the bottom of pistils, while pollen tube length of double mutants have only grown to 76.45% of pistil (Figure 4A,B). These results indicated that the pollen tube elongation of *atexpa4expb5* was significantly slower than that of wild-type after self-pollination. However, no difference was observed in *atexpa4* and *atexpb5* when compared with wild-type at various time points after pollination (Figure 4). However, the overexpression of *AtEXPA4* and *AtEXPB5* had no significant effect on pollen germination and elongation (Figure S8).

**Figure 5:**
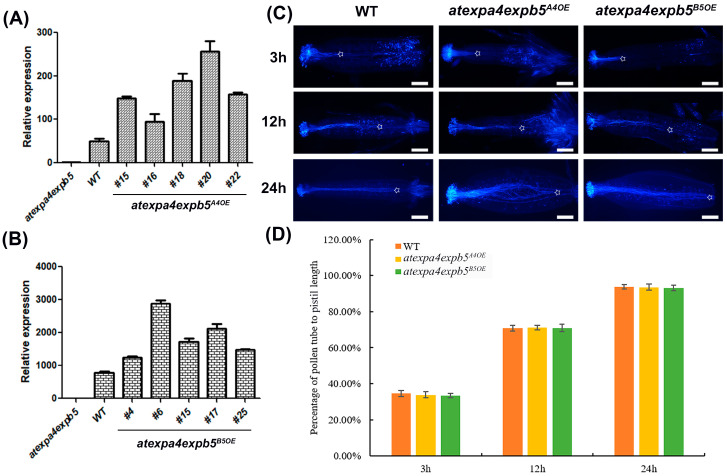
*AtEXPA4* or *AtEXPB5* can complement the slow pollen tube growth. (**A**,**B**), qRT-PCR analysis of *AtEXPA4* (**A**) or *AtEXPB5* (**B**) expression in 35-d-old inflorescences from *atexpa4expb5*, *atexpa4expb5^A4OE^,* and *atexpa4expb5^B5OE^*. *BETA-TUBULIN4* was used as the reference gene, and expression in *atexpa4expb5* was normalized to 1. The values are the mean ± SD, three biological replicates with three technical replicates in each biological replicate. (**C**) Aniline blue staining of pollen tubes at 3 h, 12 h, and 24 h after pollination, hours (h). Asterisks show positions where pollen tubes arrive, scale bars, 300 μm. (**D**) the percentage of pollen tube to pistil length in (**C**). Values are means, error bars are SD, *n* = 7 pistils per replicate, three biological replicates.

In addition, we investigated whether *AtEXPA4* or *AtEXPB5* could complement the double mutant phenotype by observing the pollen germination in vivo of complementary lines. The results of qRT-PCR showed that expression levels of *AtEXPA4* and *AtEXPB5* in corresponding complementary lines were increased (Figure 5A,B). We selected the highest expression lines *atexpa4expb5^A4OE^*-*18*/*20*/*22* and *atexpa4expb5^B5OE^*-*4*/*15*/*17* for pollen germination in vivo. The results indicated that whether transformed the double mutants with *AtEXPA4* or *AtEXPB5* could complement the slow pollen tube growth (Figure 5C,D).

Nevertheless, the silique and seed development of either two single mutants or double mutant were not different from that of wild-type (Figure S6), which implied that knocking out *AtEXPA4* and *AtEXPB5* simultaneously did not affect the transmission of sperm cells and double fertilization process. We also calculated and analyzed the segregation ratio of F_2_ plants produced by self-crossing of F_1_ plants [*expa4*(+/−) *expb5*(+/−)], and the results showed that the slower growth did not cause expansin deficient pollen to be less competitive than wild-type pollen (Table 1). In summary, *AtEXPA4* and *AtEXPB5* were redundantly involved in pollen tube germination and elongation, but showed no effect on silique and seed development.

### 3.6. AtEXPA4 Participates in Primary Root Elongation

**Figure 6:**
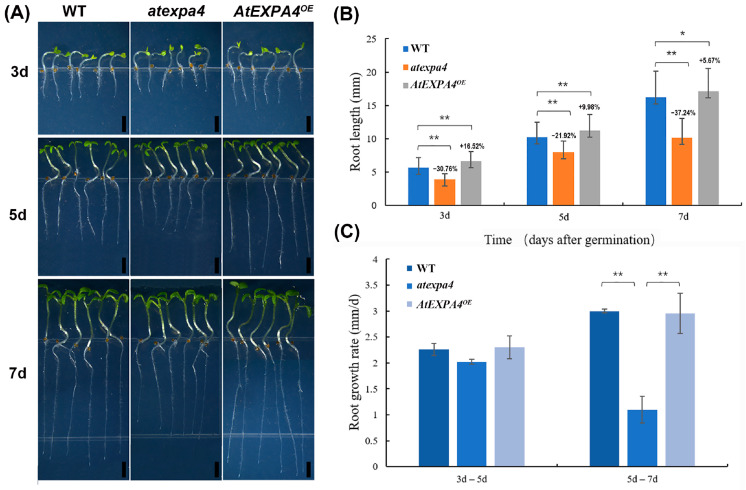
*AtEXPA4* positively regulates primary root elongation. (**A**) *AtEXPA4^OE^* and *atexpa4* seedlings germinated and grown for 3, 5, and 7 days, scale bars, 2 mm. (**B**) Root lengths in (**A**). (**C**) Root growth rates from the 3rd day to the 5th day and from the 5th day to the 7th day after seed germination. Values are means, error bars are SD, *n* ≥ 25 seedlings per replicate, three biological replicates, *t*-tests as ** *p* < 0.01 and * *p* < 0.05.

**Figure 7:**
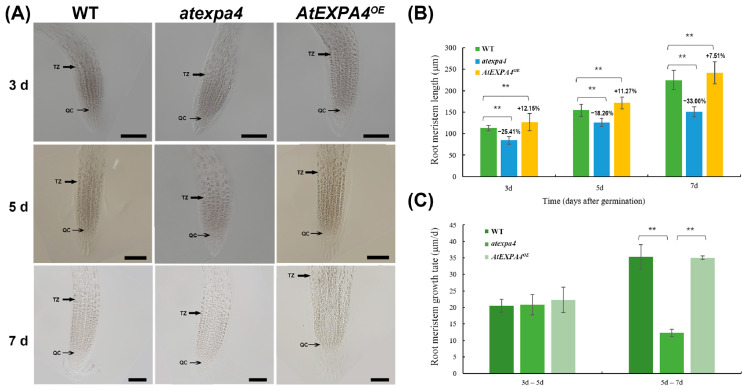
*AtEXPA4* positively regulates root meristem length. (**A**) Differential interference images of *AtEXPA4^OE^* and *atexpa4* root meristem boundary after seed germinated for 3, 5, and 7 days, scale bars, 100 μm. (**B**) Root meristem lengths in (**A**). (**C**) Root meristem growth rates from the 3rd day to the 5th day and from the 5th day to the 7th day after seed germination. Values are means, error bars are SD, *n* ≥ 25 seedlings per replicate, three biological replicates, *t*-tests as ** *p* < 0.01.

The increasing expression of *AtEXPA4* in primary roots during seedling development suggested that it might be involved in root growth. Therefore, we measured the primary root length of seedlings. Interestingly, when compared with the wild-type at 3d, 5d, and 7d after seed germination, the length of *atexpa4* primary root decreased by 30.76%, 21.92%, and 37.24%, respectively. On the contrary, *AtEXPA4^OE^* seedlings exhibited increased primary root length by 16.52% and 9.98% at 3d and 5d after seed germination. Although the primary root length of *AtEXPA4^OE^* only increased by 5.67% compared with the wild-type at the 7th day after germination, it still reached a significant level. (Figure 6A,B). Moreover, the mutation and overexpression of *AtEXPB5* did not affect the growth and development of roots (Figure S9A,B). Notably, the change pattern of root meristem size was similar to that of root length. The meristem length of *atexpa4* was reduced by 25.41%, 18.26%, and 33.00% compared with the wild-type after 3, 5, and 7 days of seed germination (Figure 7A,B). By contrast, the overexpression of *AtEXPA4* led root meristems to increase by 12.15%, 11.27%, and 7.51% at 3d, 5d, and 7d after seed germination (Figure 7A,C).

In addition, the primary root growth rates of wild-type, *atexpa4*, and *AtEXPA4^OE^* were similar from the 3rd day to the 5th day after germination (Figure 6C). However, some changes occurred from the 5th day to the 7th day after germination. On the one hand, primary root growth rates of wild-type and *AtEXPA4^OE^* were still consistent but higher than before. On the other hand, the primary root growth rate of *atexpa4* was slower than that of wild-type and *AtEXPA4^OE^* (Figure 6C). The change pattern of meristem growth rate was consistent with that of root elongation rate. That is, the meristem growth rate of each transgenic line was closely resembled in the first 5 days after seed germination, while the growth rate of *atexpa4* was significantly reduced from the 5th day to the 7th day (Figure 7A,C). These findings suggested that *AtEXPA4* might exert the greatest effect from the 5th day to the 7th day after seed germination. For *AtEXPB5*, the primary root growth rates of *atexpb5* and *AtEXPB5^OE^* were not significantly different from that of wild-type (Figure S9C). Taken together, *AtEXPA4* could promote the elongation of primary root and root meristem during root growth and development, but *AtEXPB5* had no effect on the growth of primary root.

## 4. Discussion

During pollen tube growth, cell walls provide mechanical strength resisting turgor pressure to protect two sperm cells [46]. Numerous cell wall synthesis and remodeling genes have been reported in this process. In terms of cell wall synthesis, mutations of pollen-expressed *A. thaliana* cellulose synthase-like D genes *CSLD1* and *CSLD4* caused significant reduction in cellulose deposition of pollen tube wall, which disrupted the genetic transmission of male gametophytes [3]. Moreover, two putative *A. thaliana* galacturonosyltransferase genes *GAUT13* and *GAUT14* were essential for pollen tube growth [47]. Changes in cell wall assembly are relevant to pollen tube mechanical properties. For example, suppressing the expression of four leucine-rich repeat extensin genes (*LRX8–11*) compromised pollen germination and pollen tube growth [46,48]. In terms of cell wall remodeling, pectin methylesterase (PME) demethylates pectin to maintain stable growth of pollen tubes. Mutations of *PME* genes, such as *VGD1* [9], *AtPPME1* [49], and *AtPME48* [6], showed retarded pollen tube growth in vivo and *in vitro*. Additionally, PME inhibitors (PMEIs) are thought to be key regulators of cell wall stability at the tip of pollen tube. Suppressing the expression of *Brassica oleracea PMEI1* resulted in partial male sterility and decreased seed set by inhibition of pollen tube growth [50]. Furthermore, polygalacturonase, which leads to degradation of pectin and decomposition of cell walls, has also been confirmed to play an important role in the development of pollen tubes [7,51]. Expansins, as a type of cell wall remodeling proteins, can respond to the rapid expansion of cell walls and affect pollen tube growth [52]. Our results confirmed that the expressions of *AtEXPA4* and *AtEXPB5* were significantly high in mature pollen grains and pollen tubes. Moreover, proteins coding by these two genes were located on the cell wall and in the cytoplasm, and this was consistent with the positioning result of a soybean expansin protein GmEXPB2 [53]. Two single mutants, *atexpa4* and *atexpb5*, did not exhibit any observable defects in pollens and pollen tubes, but *atexpa4expb5* mutant was defective in pollen tube elongation. Moreover, the results of complementary experiments showed that whether transformed the double mutants with *AtEXPA4* or *AtEXPB5* could complement the slow pollen tube growth. However, the overexpression of *AtEXPA4* and *AtEXPB5* did not affect pollen tube growth. We speculate that this may be because the highest multiple of *AtEXPA4^OE^* and *AtEXPB5^OE^* overexpression line does not exceed 40 folds, which may not be enough to accelerate the rate of pollen tube elongation. Additionally, although the pollen germination rate of the double mutants decreased, the mutation of AtEXPA4 and AtEXPB5 did not affect the pollen competitiveness or the normal development of siliques. Taken together, *AtEXPA4* and *AtEXPB5* only showed redundant functions in pollen tube growth.

Interestingly, there are abundant MYB binding sites on promoters of *AtEXPA4* and *AtEXPB5* (Figure S10), which indicates that MYB transcription factors may participate in pollen tube and root growth by regulating expressions of *AtEXPA4* and *AtEXPB5*. A previous study showed that a R2R3 MYB factor, TDF1, affected tapetum development by directly binding to *AtEXPB5* promoter and co-regulated *AtEXPB5* with another transcription factor AMS [54]. Thence, *AtEXPB5* might play a role in tapetum and pollen exine development. However, there was no defect in exine of *atexpb5* pollens, suggesting that TDF1 regulated the development of tapetum by acting on other cell wall remodeling genes. Pollen tube germination usually starts from the intine of mature pollen grains [6]. *AtEXPA4* and *AtEXPB5* were strongly expressed in mature pollen grains and pollen tubes, and simultaneous mutation of them led to obstruction of pollen tube elongation, implying that they might affect pollen tube growth by regulating the development of pollen intine. Moreover, *AtMYB4*, *AtMYB32*, *AtMYB97*, *AtMYB101*, and *AtMYB120* can participate in pollen and pollen tube development [55,56], suggesting that they may regulate expressions of *AtEXPA4* and *AtEXPB5*, but further evidence is needed.

Some cell wall-related genes that affect pollen tube growth also play roles in root elongation. For example, a rhamnogalacturonan II xylosyltransferase (RG-II) gene, *MGD4*, participated in the growth of pollen tube and root by acting on pectic RG-II biosynthesis pathway [5]. In this study, qRT-PCR and *GUS* analyses also showed that *AtEXPA4* was highly expressed in roots. Furthermore, it was confirmed that *AtEXPA4* affected primary root elongation positively by comparing the primary root length of *atexpa4* and *AtEXPA4^OE^* at different time points after seed germination. In addition, the growth rate of *atexpa4* primary root sharply decreased from the 5th day to the 7th day after germination, suggesting that *AtEXPA4* exerted the greatest effect during this period. We speculated that this effect might be related to the gradual increase of *AtEXPA4* expression with the growth and development of primary roots. (Figure 1D). However, the mutation and overexpression of *AtEXPB5* did not affect the growth and development of the primary roots (Figure S9). This indicates that *AtEXPA4* plays a major role in the growth and development of primary roots, and *AtEXPB5* does not affect root growth because of its extremely low expression in roots. Generally, abnormal root system also affects the response of plants to abiotic stress. The overexpression of *Triticum aestivum EXPB23* (*TaEXPB23*) showed increased lateral roots and higher root biomass, as well as enhanced drought tolerance [57]. Additionally, the analysis of *AtEXPA4* and *AtEXPB5* promoters showed that they both contained abundant response elements of abscisic acid, ethylene, and jasmonic acid (Figure S10), suggesting that they might participate in plant responses to abiotic stress. Further research is needed to confirm this.

## 5. Conclusions

We isolated and characterized two expansin genes, *AtEXPA4* and *AtEXPB5*, which are strongly expressed in mature pollens and pollen tubes. The molecular functions of *AtEXPA4* and *AtEXPB5* were analyzed by CRISPR/Cas9-mediated knockout and self-promoter-mediated overexpression. The results indicated that *AtEXPA4* and *AtEXPB5* are redundantly required for pollen tube growth. This enriches the role of expansins in the reproductive development of plants, and also shows that the genes of EXPA and EXPB subfamily can coordinate with each other to regulate plant growth and development. Furthermore, *AtEXPA4* also showed a higher expression level in roots. Based on the statistics of primary root length and root meristem size, we found that *AtEXPA4* has a positive effect on the growth of primary roots. This also provides evidence for the involvement of expansin in the growth and development of plant roots. In addition, since there are many MYB binding sites and a variety of phytohormone response elements in the promoters of *AtEXPA4* and *AtEXPB5*, future research can start from these aspects to uncover the regulatory pathways of expansin genes on plant growth and development.

## Figures and Tables

**Table 1:** Segregation data from self-crossing of heterozygous F_1_ double mutants [*expa4*(+/−) *expb5*(+/−)].

Genotype of F_2_ Plants	The Observed Number of F_2_ Plants	The Expected Number of F_2_ Plants	Degrees of Freedom	χ^2^ Test for 9:3:3:1 ^a^	
				χ^2^	Expected χ^2^ (*p* < 0.05)
*expa4*(+/) *expb5*(+/)	196	203.625	3	4.576 ^b^	7.815
*expa4*(−/−) *expb5*(+/)	76	67.875			
*expa4*(+/) *expb5*(−/−)	75	67.875			
*expa4*(−/−) *expb5*(−/−)	15	22.625			

^a^ χ^2^ tests were conducted to test a null hypothesis that F_2_ plants segregate from each other in a predicted 9:3:3:1 Mendelian ratio. ^b^ χ^2^ < Expected χ^2^ indicated that the null hypothesis was accepted, that is, the separation ratio of F_2_ plants accorded with the 9:3:3:1 Mendelian ratio.

## Data Availability

Data sharing not applicable.

## Supplementary Materials

The following are available online at https://www.mdpi.com/2073-4425/12/2/249/s1, Figure S1: Amino acid sequences of AtEXPA4 and AtEXPB5. The blue, red, and green letters represent signal peptide, DPBB_1 domain, and Pollen_allerg_1 domain, respectively. Figure S2: Subcellular localizations of AtEXPA4::eGFP and AtEXPB5::eGFP fusion proteins in tobacco leaf epidermal cells. Figure S3: Structures of CRISPR/Cas9 (**A**) and *AtEXPA4* (**B**) *AtEXPB5* (**C**) overexpression binary vectors for *Arabidopsis thaliana* transformation by floral dip method. The hSpCas9 cassette is driven by *YAO* promoter, while sgRNAs are controlled by *AtU6-26* promoters. NLS, nuclear localization sequence. Figure S4: Sequence alignment of *atexpa4* and *atexpb5* mutants showed evidence of successful gene editing in the target regions, respectively. Figure S5: Sequence alignment of *atexpa4expb5* mutants showed that the lines with both *AtEXPA4* and *AtEXPB5* knockout sites were successfully screened. (**A**), Detection of editing sites of *AtEXPA4*. (**B**) Detection of editing sites of *AtEXPB5*. Figure S6: Morphological observation of transgenic plants and floral organs. The first row is the overall shape of transgenic plants, scale bars, 4 cm, and the upper right corners are corresponding siliques, scale bars, 4 mm. The second row is the overall shape of flowers. The third row is the shape of pistils and stamens, scale bars, 1mm. Figure S7: Pollen morphology in *atexpa4*, *atexpb5*, *atexpa4expb5*, *AtEXPA4^OE^*, and *AtEXPB5^OE^*. Cytological staining observation includes Alexander staining, scale bars, 100 μm, DAPI staining, scale bars, 50 μm, and aniline blue staining, scale bars, 50 μm. SEM is used to observe pollen grain morphology and surface decoration, scale bars, 40 μm. Values are means, error bars are SD, *n* = 12 pistils per replicate, three biological replicates. Figure S8: Overexpression of *AtEXPA4* and *AtEXPB5* did not affect pollen elongation. (**A**) Aniline blue staining of pollen tubes at 3 h, 12 h, and 24 h after pollination in *AtEXPA4^OE^* lines and *AtEXPB5^OE^* lines, hours (h). Asterisks show positions where pollen tubes arrive, scale bars, 300 μm. (**B**) the percentage of pollen tube to pistil length in (**A**). Values are means, error bars are SD, *n* = 12 pistils per replicate, three biological replicates. Figure S9: *AtEXPB5* does not affect the primary root elongation. (**A**) *AtEXPB5^OE^* and *atexpb5* seedlings germinated and grown for 3, 5, and 7 days, scale bars, 2 mm. (**B**) Root lengths in (**A**). (**C**) Root growth rates from the 3rd day to the 5th day and from the 5th day to the 7th day after seed germination. Values are means, error bars are SD, n ≥ 12 seedlings per replicate, three biological replicates. Figure S10: Distribution of *cis*-acting elements in the 1.5 kb upstream promoter regions of *AtEXPA4* and *AtEXPB5*. The different types of *cis*-acting elements are represented by different colors. The scale above was to measure nucleotides length. The results are predicted by the PlantCARE website (http://bioinformatics.psb.ugent.be/webtools/plantcare/html/). Table S1. Primers used in this study. Table S2. Off-target detection of *atexpa4*. Table S3. Off-target detection of *atexpb5*.
