# Supplementary material for: Two Expansin Genes, AtEXPA4 and AtEXPB5, Are Redundantly Required for Pollen Tube Growth and AtEXPA4 Is Involved in Primary Root Elongation in Arabidopsis thaliana
Source: Genes (Basel). 2021 Feb 10;12(2):249. doi: 10.3390/genes12020249 (PMC7916401; doi:10.3390/genes12020249)
Supplement: Supplementary file 1 [file genes-12-00249-s001.zip › Supplementary files_Revision/Supplementary Figures 1-10_revision.docx]

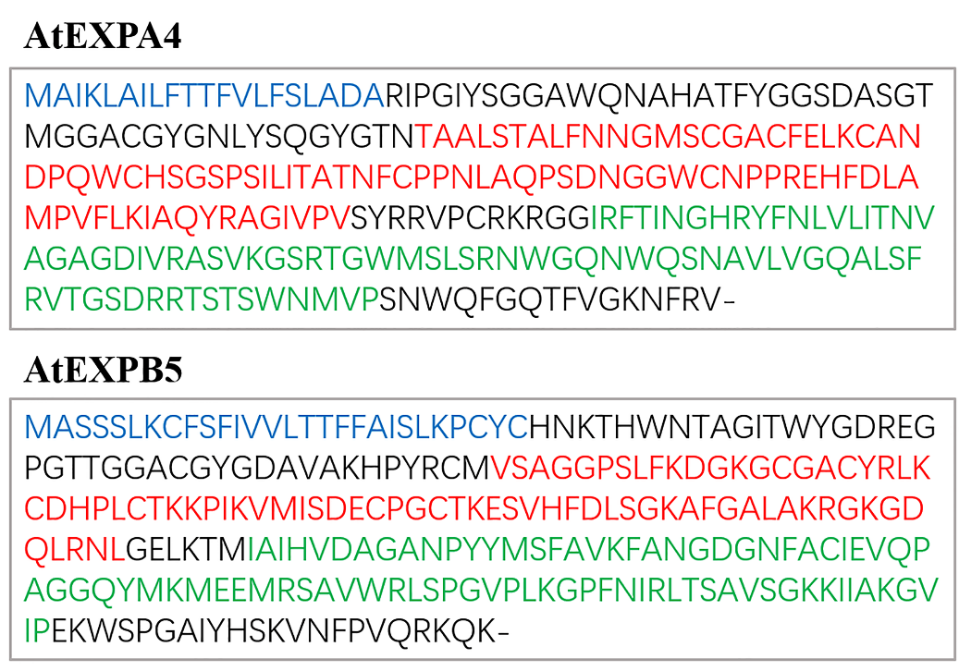


**Figure S1. Amino acid sequences of AtEXPA4 and AtEXPB5.** The blue, red, and green letters represent signal peptide, DPBB_1 domain, and Pollen_allerg_1 domain, respectively.


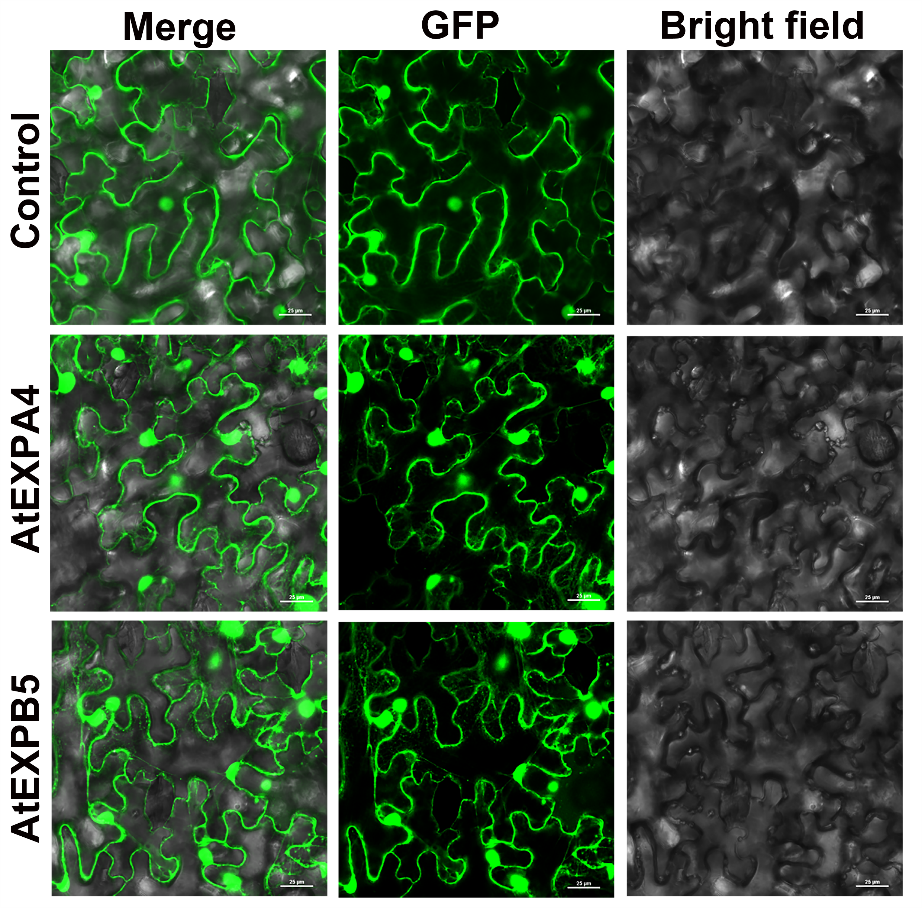


**Figure S2. Subcellular localizations of AtEXPA4::eGFP and AtEXPB5::eGFP fusion proteins in tobacco leaf epidermal cells.** The first row is control cells with eGFP signals. The second row is leaf epidermal cells with AtEXPA4::eGFP fusion signals. The last row is leaf epidermal cells with AtEXPB5::eGFP fusion signals. Scale bars, 25 μm.


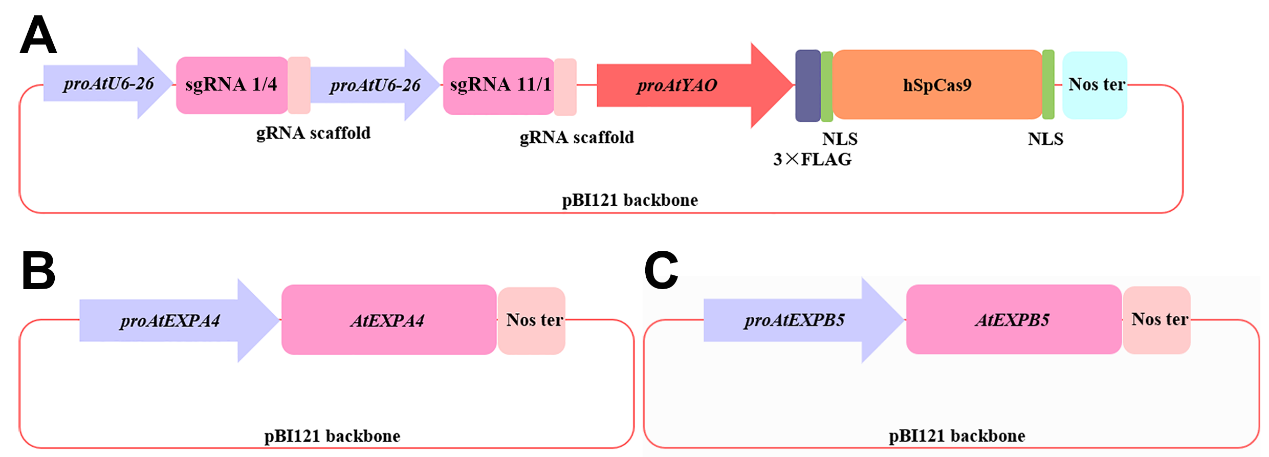


**Figure S3. Structures of CRISPR/Cas9 (A) and *AtEXPA4* (B), *AtEXPB5* (C) overexpression binary vectors for *Arabidopsis thaliana* transformation by ﬂoral dip method.** The hSpCas9 cassette is driven by *YAO* promoter, while sgRNAs are controlled by *AtU6-26* promoters. NLS, nuclear localization sequence.


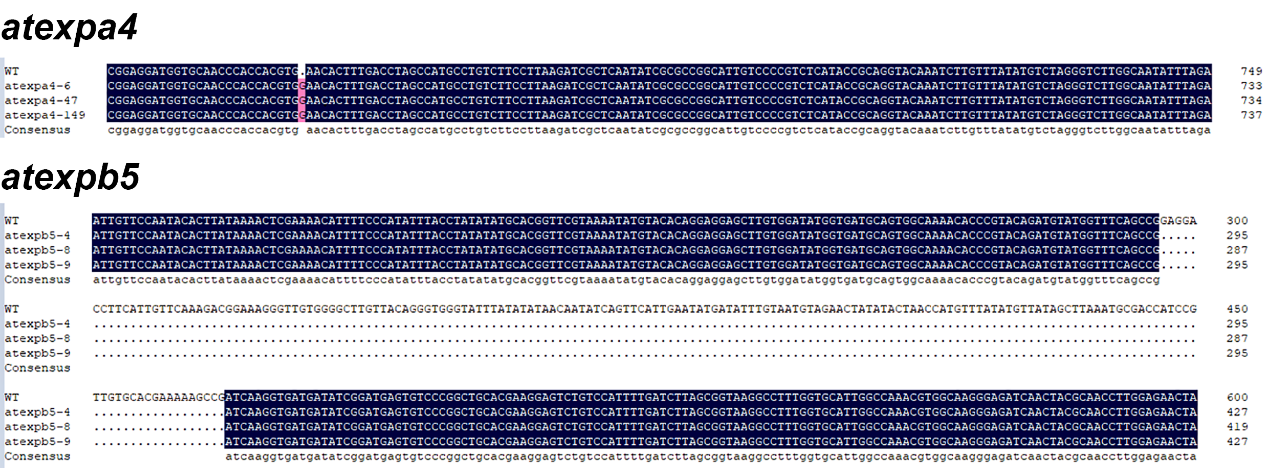


**Figure S4. Sequence alignment of *atexpa4* and *atexpb5* mutants** **showed evidence of successful gene editing in the target regions, respectively.**


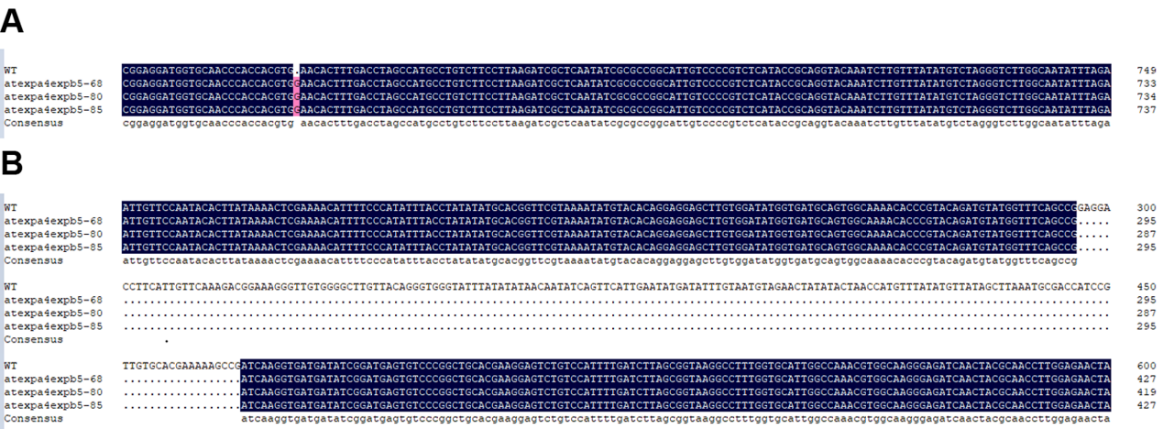


**Figure S5. Sequence alignment of *atexpa4expb5* mutants showed that the lines with both *AtEXPA4* and *AtEXPB5* knockout sites were successfully screened.** A, Detection of editing sites of *AtEXPA4*. B, Detection of editing sites of *AtEXPB5*.


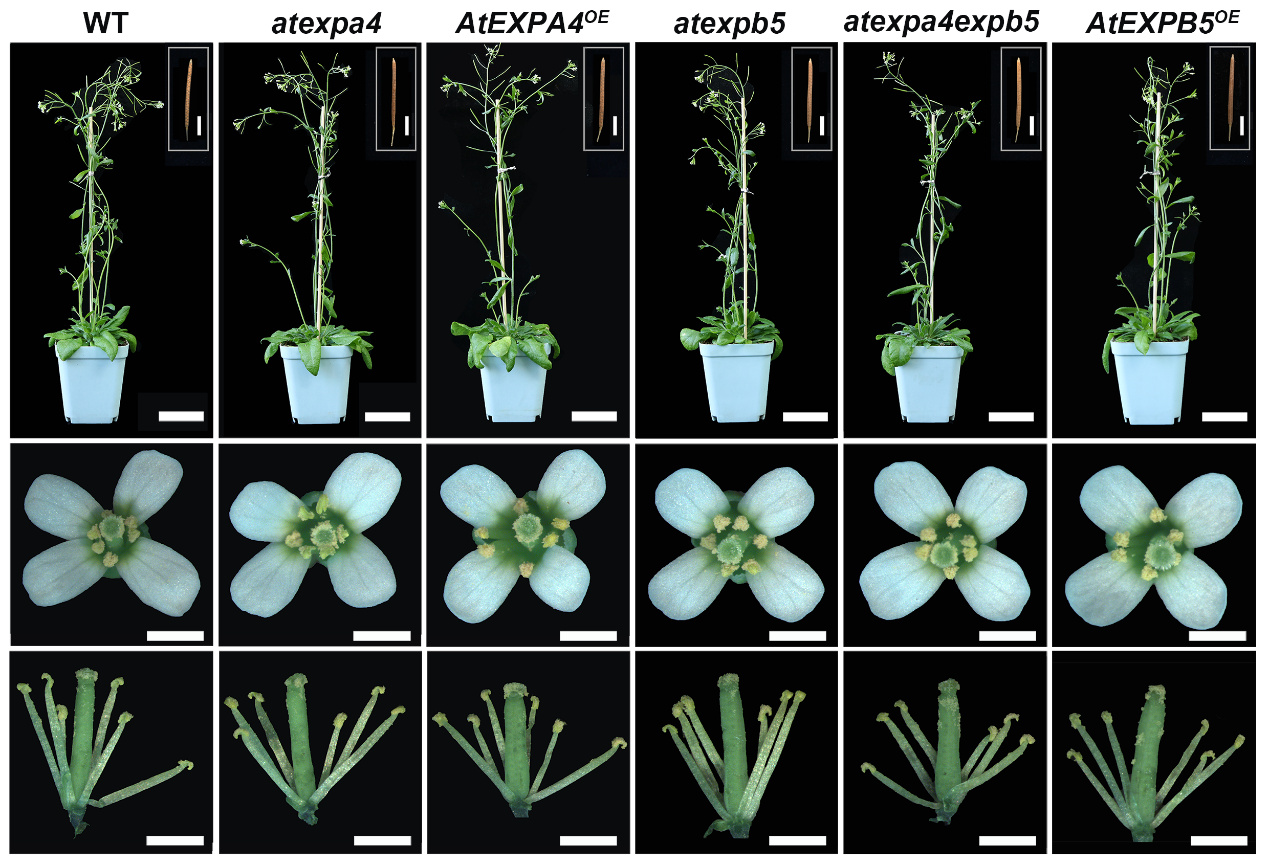


**Figure S6.** **Morphological observation of transgenic plants** **and floral organs.** The first row is the overall shape of transgenic plants, scale bars, 4 cm, and the upper right corners are corresponding siliques, scale bars, 4 mm. The second row is the overall shape of flowers. The third row is the shape of pistils and stamens, scale bars, 1mm.


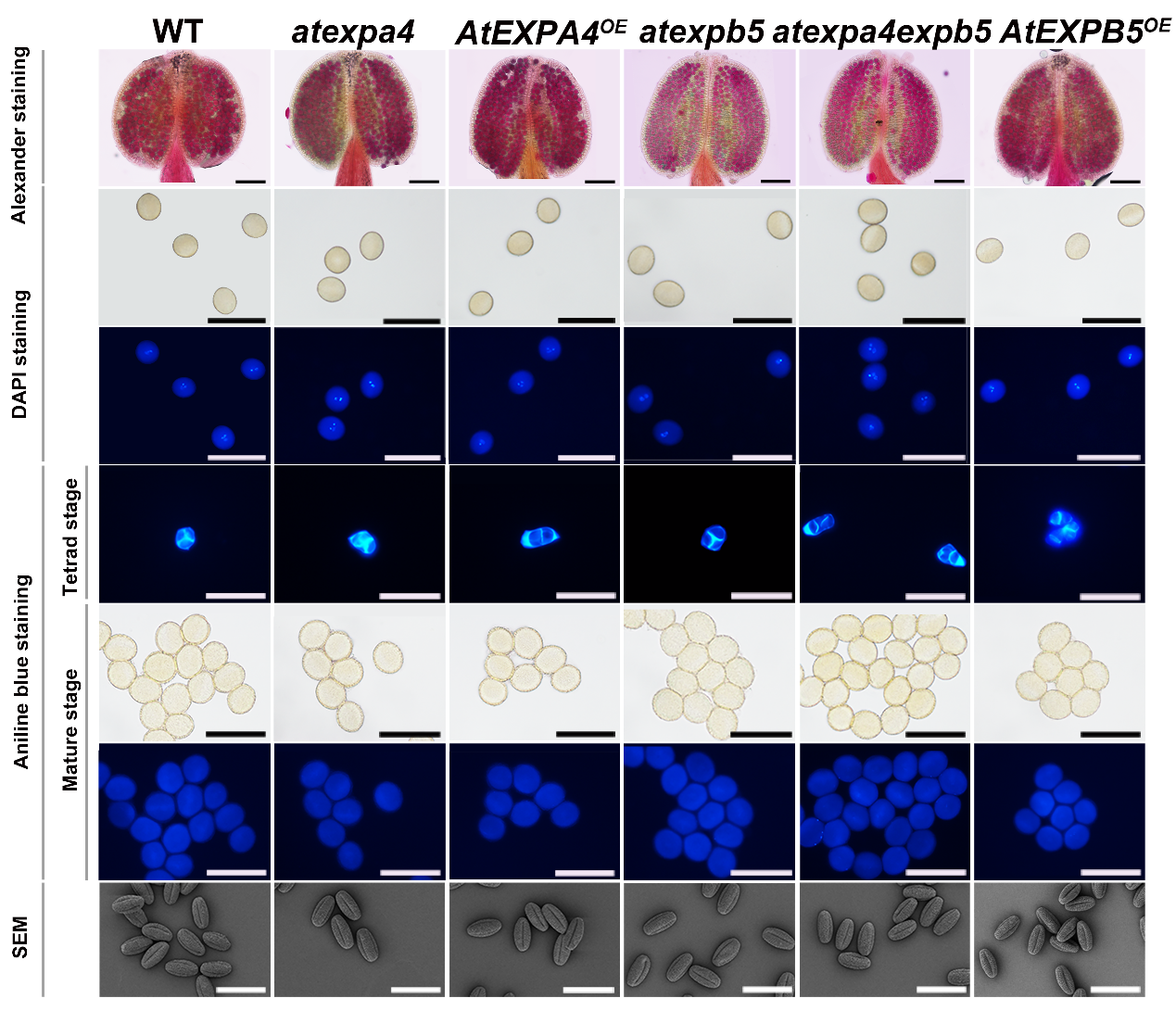


**Figure S7. Pollen morphology in *atexpa4*, *atexpb5*, *atexpa4expb5*, *AtEXPA4^OE^*, and *AtEXPB5^OE^*.** Cytological staining observation includes Alexander staining, scale bars, 100 μm, DAPI staining, scale bars, 50 μm, and aniline blue staining, scale bars, 50 μm. SEM is used to observe pollen grain morphology and surface decoration, scale bars, 40 μm.


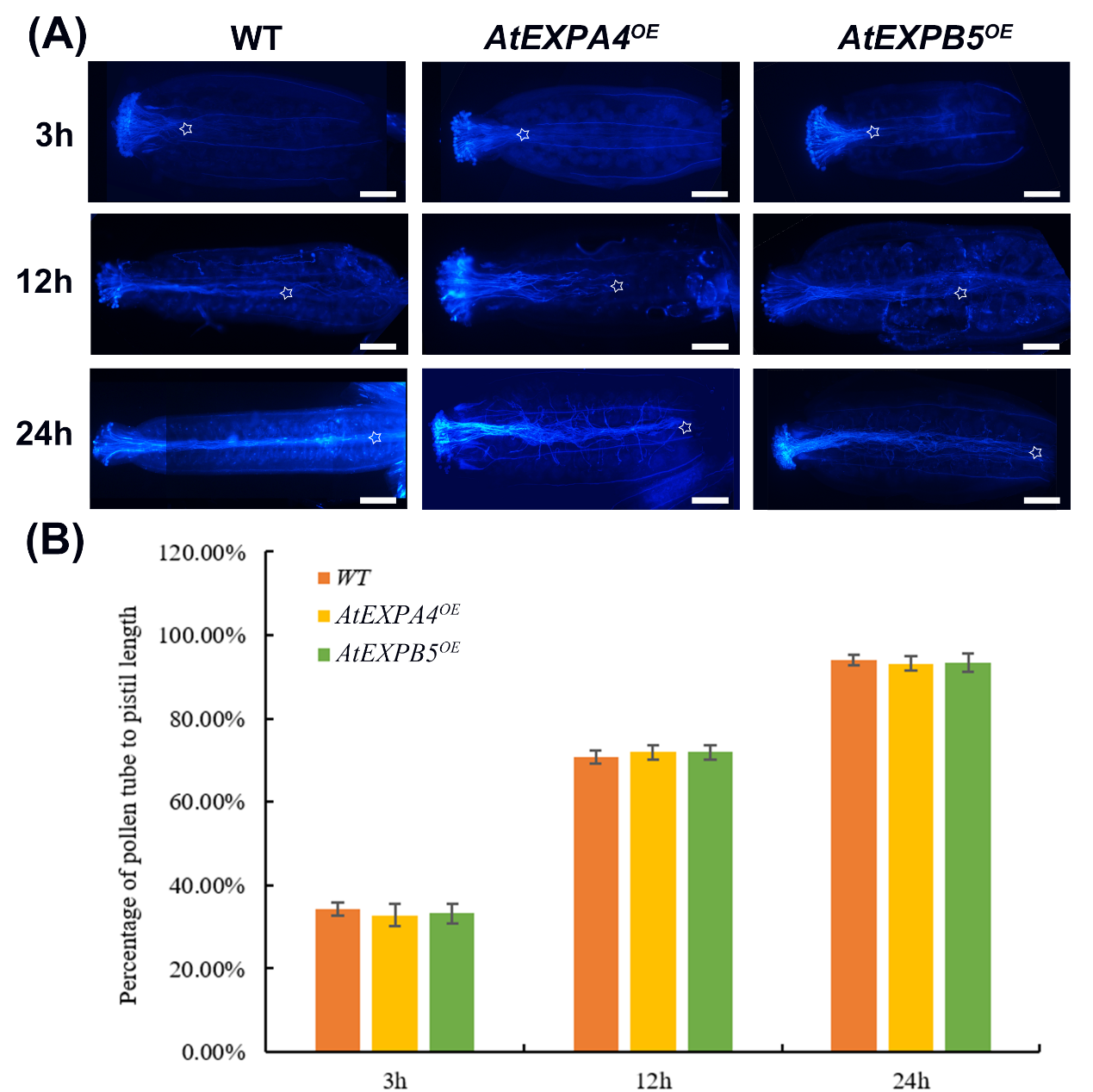


**Figure S8.** Overexpression of *AtEXPA4* and *AtEXPB5* did not affect pollen elongation. A, Aniline blue staining of pollen tubes at 3 h, 12 h, and 24 h after pollination in *AtEXPA4^OE^* lines and *AtEXPB5^OE^* lines, hours (h). Asterisks show positions where pollen tubes arrive, scale bars, 300 μm. B, the percentage of pollen tube to pistil length in (A). Values are means, error bars are SD, n = 12 pistils per replicate, three biological replicates.


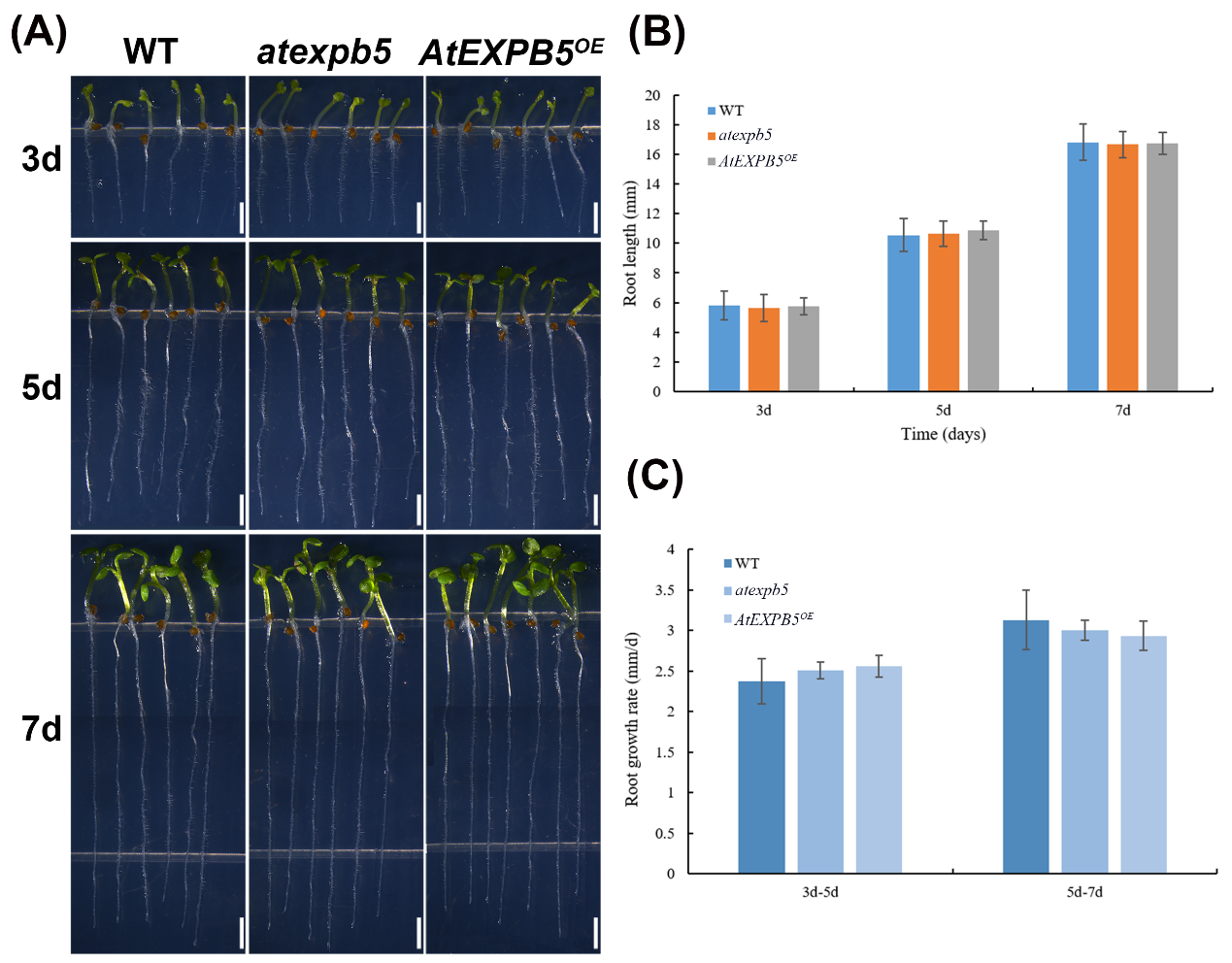


**Figure S9. *AtEXPB5* does not affect the primary root elongation.** A, *AtEXPB5^OE^* and *atexpb5* seedlings germinated and grown for 3, 5, and 7 days, scale bars, 2 mm. B, Root lengths in (A). C, Root growth rates from the 3^rd^ day to the 5^th^ day and from the 5^th^ day to the 7^th^ day after seed germination. Values are means, error bars are SD, n ≥ 12 seedlings per replicate, three biological replicates.


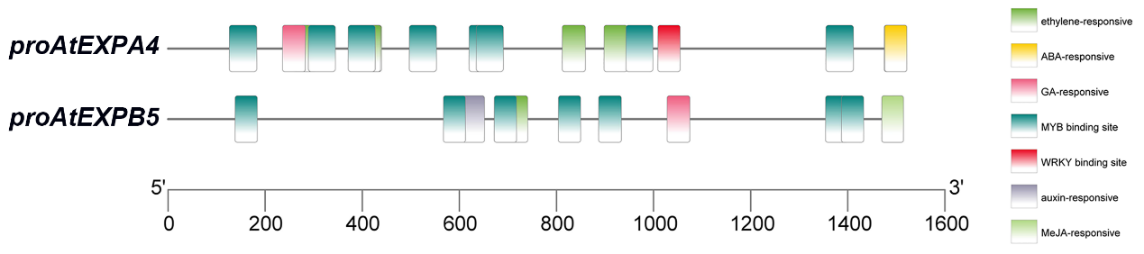


**Figure S10. Distribution of *cis*-acting elements in the 1.5 kb upstream promoter regions of *AtEXPA4* and *AtEXPB5*.** The different types of *cis*-acting elements are represented by different colors. The scale above was to measure nucleotides length. The results are predicted by the PlantCARE website (http://bioinformatics.psb.ugent.be/webtools/plantcare/html/).
