# Supplementary material for: Two Expansin Genes, AtEXPA4 and AtEXPB5, Are Redundantly Required for Pollen Tube Growth and AtEXPA4 Is Involved in Primary Root Elongation in Arabidopsis thaliana
Source: Genes (Basel). 2021 Feb 10;12(2):249. doi: 10.3390/genes12020249 (PMC7916401; doi:10.3390/genes12020249)
Supplement: Supplementary file 1 [file genes-12-00249-s001.zip › Supplementary files_Revision/Supplementary Tables 1-3_revision.docx]

**Table S1. Primers used in this study**

| **Application** | **Primer name** | **Primer Sequence (5'-3')** |
| --- | --- | --- |
| CDS sequence amplification | AtEXPA4-CDS-F1 | ATGGCTATTAAACTAGCAATTCTAT |
|  | AtEXPA4-CDS-R1 | CCTGAAATTCTTCCCGACA |
|  | AtEXPB5-CDS-F1 | ATGGCTTCCTCATCTCTCAAATGTTT |
|  | AtEXPB5-CDS-R1 | TCATTTCTGCTTCCTTTGAACGG |
| Promoter sequence amplification | proAtEXPA4-F1 | AAGTAAAGAAATGTTGAATAGTGG |
|  | proAtEXPA4-R1 | TTTGTGTGTGAATTACTAGAAACAG |
|  | proAtEXPB5-F2 | CTAACTGATCGGTACAGAACCAAGA |
|  | proAtEXPB5-R2 | TGAATGGGTAGAGATTTTTTAGAGC |
| qRT-PCR | qRT-Tubulin-F | AAGGCTTTCCTTCATTGGTACA |
|  | qRT-Tubulin-R | CTCTCCGGCTGTAGCATCTT |
|  | qRT-AtEXPB5-F1 | TCAAAGACGGAAAGGGTTGTGGG |
|  | qRT-AtEXPB5-R1 | CTTTTAGTTCTCCAAGGTTGCGTAGTT |
|  | qRT-AtEXPA4-F2 | CAATGGTATGAGTTGTGGAGCC |
|  | qRT-AtEXPA4-R2 | GGACTACCTGAGTGACACCATTGA |
| Detection of mutants | AtEXPB5_ko_check-F1 | ATGTTTTTCTTTTATCGTTGTTCTT |
|  | AtEXPB5_ko_check-R1 | CGAATGACATGTAGTAAGGATTTG |
|  | AtEXPA4_ko_check-F1 | TCATCTCTCAATCAACAAACCTAAC |
|  | AtEXPA4_ko_check-R1 | TATCATCAAAATTTGATTTTCAAGG |
| Off-target detection | AtEXPA4-Targetoff1-F1 | GGTGCTTGTGGATATGGTAATCTAT |
|  | AtEXPA4-Targetoff1-R1 | CTGTAGGAAACAGGGACGATTC |
|  | AtEXPA4-Targetoff2-F1 | GTGTGGGTACGGGAATTTGTATAGC |
|  | AtEXPA4-Targetoff2-R1 | CCTGCGGTAAGAGACGGGGA |
|  | AtEXPB5-Targetoff1-F2 | TGTCATCGCTCCTGCCTGTC |
|  | AtEXPB5-Targetoff1-R2 | GGAAACATGGAGTAGTTGGAAAGTT |
|  | AtEXPB5-Targetoff2-F3 | ATGATTCATTACCTGGAGTGT |
|  | AtEXPB5-Targetoff2-R3 | ATCTAGGGTTTCTGTTTCTTG |
| Detection of overexpression plants and restored lines | AtEXPA4_OE_check_F1 | ATGGCTATTAAACTAGCAATTCTAT |
|  | AtEXPA4_OE_check_R1 | ACACAGGAAACAGCTATGACC |
|  | AtEXPB5_OE_check_F1 | ATGGCTTCCTCATCTCTCAAATGTTT |
|  | AtEXPB5_OE_check_R1 | ACACAGGAAACAGCTATGACC |

**Table S2. Off-target detection of *atexpa4***

| **Site** | **Gene** | **Region** | **Sequence** | ***atexpa4-6*** | ***atexpa4-47*** | ***atexpa4-149*** |
| --- | --- | --- | --- | --- | --- | --- |
| Target_off_1 | At1g26770 | exon | GCAAGGTCAAAGTGTTCAAGAGG | 0 (11) | 0 (11) | 0 (11) |
| Target_off_2 | At5g02260 | exon | GCGAGATCAAAGTGCTCACGAGG | 0 (11) | 0 (11) | 0 (11) |

PAM sequences are highlighted in red. The total number of plants checked is shown in parentheses and the number before the parentheses indicates off-target plants.

**Table S3. Off-target detection of *atexpb5***

| **Site** | **Gene** | **Region** | **Sequence** | ***Atexpb5-4*** | ***Atexpb5-8*** | ***Atexpb5-9*** |
| --- | --- | --- | --- | --- | --- | --- |
| Target_off_1 | At4g35830 | exon | GCTTTTTCATACACAGGGGAAGG | 0 (13) | 0 (15) | 0 (19) |
| Target_off_2 | At4g30120 | exon | GCTGCAACCATAAATCTTAATGG | 0 (13) | 0 (15) | 0 (19) |

PAM sequences are highlighted in red. The total number of plants checked is shown in parentheses and the number before the parentheses indicates off-target plants.
